# SARS‐CoV‐2 Chronic Intervillositis: Variations of Maternal Antiviral Response

**DOI:** 10.1111/aji.70215

**Published:** 2026-02-09

**Authors:** Daria Kozlova, Ori Mayer, Noam Shomron, Yosef Azan, Rani Shlayem, Yevgeni Yegorov, Nir Rainy, Avi Natan, Ana Foigelman Tobar

**Affiliations:** ^1^ Department of Pathology Rabin Medical Center – Beilinson Hospital Petach Tikva Israel; ^2^ Faculty of Medicine Tel Aviv University Tel Aviv Israel; ^3^ Shamir Medical Center (Assaf Harofeh) Zrifin Israel

**Keywords:** CD20, chronic histiocytic intervillositis, maternal inflammation, placenta, SARS‐CoV‐2

## Abstract

**Problem:**

Placental compromise is a determining factor in the outcome of pregnancies complicated by maternal SARS‐CoV‐2 infection. Chronic histiocytic intervillositis (CHI) is the initial response to SARS‐CoV‐2 infection, but the underlying mechanisms are poorly understood. The aim of this study was to assess the extent and composition of the placental inflammatory response and to explore the relationship between the severity of inflammation and viral levels in the placenta.

**Methods of study:**

Placentas from 43 women who tested positive for SARS‐CoV‐2 infection at the time of delivery at a single tertiary medical center (8/2020 to 10/2022) were evaluated using quantitative reverse transcription polymerase chain reaction (qRT‐PCR), histopathological, ultrastructural, and in situ hybridization studies.

**Results:**

There was a significant association between viral load and severity of CHI with notable involvement of CD20‐positive B lymphocytes (100% in patients with diffuse CHI vs 0% in the patients with no inflammation). Both PCR‐positive and PCR‐negative placentas exhibited varying degrees of inflammation. Electron microscopy confirmed viral particles in PCR‐ and CHI‐negative samples, Additionally, mean gestational age at delivery was significantly lower in the PCR‐positive patients.

**Conclusion:**

These findings highlight the complex interplay between maternal SARS‐CoV‐2 infection and placental inflammation. The inflammatory response may extend beyond the presence of the virus per se, and viral load differences linked to individual immune response variability may influence the development of inflammation. Further research is needed to elucidate the mechanisms by which SARS‐CoV‐2 impacts maternal and neonatal health outcomes and provide insight into the implications of viral infections during pregnancy.

## 1 Introduction

Pregnant women infected with severe acute respiratory syndrome coronavirus 2 (SARS‐CoV‐2) are at increased risk of maternal and fetal morbidities [1]. One of the main factors affecting outcome is placental compromise. Various histopathological changes in the placenta have been described in the setting of SARS‐CoV‐2 infection, including maternal and fetal vascular malperfusion, massive perivillous fibrin deposition, and inflammatory lesions, namely chronic chorioamnionitis, chronic deciduitis, chronic villitis, and chronic histiocytic intervillositis (CHI). The unique pattern of CHI and/or massive perivillous fibrin deposition has been coined SARS‐CoV‐2 placentitis [2, 3].

CHI is the initial response to SARS‐Cov‐2 infection and may be associated with a risk of vertical transmission of the virus. It. has been suggested that angiotensin‐converting enzyme 2 (ACE2) receptors found on syncytiotrophoblasts serve as the main mechanism of entry of the virus. The process is mediated by endocytosis, supported by a host of transmembrane proteins including the serine proteases. The infection triggers a direct and indirect immunological response [3] with the massive infiltration of a host of maternal immune cells, including CD68‐positive macrophages (Hofbauer cells), CD3‐positive and CD8‐positive T lymphocytes, and CD20‐positive B cells. This leads to an upregulation of the proinflammatory cytokines interleukin (IL)‐6, interferon‐induced protein 10 (IP10), and monokine induced by interferon gamma (MIG) [4]. Studies focused on the role of B‐cells have shown that the disruption in B‐cell responses, especially CD20‐positive cells, exacerbates inflammation and adversely impacts pregnancy outcome [5]. However, it is still unclear why CHI develops in some patients with SARS‐CoV‐2 infection but not others.

In Israel, awareness of coronavirus disease of 2019 the disease began well before it was declared a global epidemic by the World Health Organization in March 2020. A national emergency was declared already at the end of January 2020, and over the next two months, restrictions on international travel and mass gatherings were enforced. In March 2020, following establishment of community transmission, the first lockdown was imposed to “flatten the curve”. Restrictions were lifted on April 19, 2020, but there followed a marked surge of the disease across the country, and by August 2020, more than 88,000 individuals had been affected, with about 700 deaths. Therefore, a second lockdown was imposed during September–October 2020 [6, 7].

The epidemiological landscape of SARS‐CoV‐2 in Israel underwent distinct transitions, with the emergence and dominance of specific viral variants. The Alpha variant, identified in October 2020, predominated to April 2021, and between April and December 2021, the Delta variant was the primary strain. Beginning in December 2021, there was a shift in the country to Omicron and its sublineages BA.1, BA.2, BA.5, B.Q1, XBB1.5, and XBB1.9. This succession of variants reflected the global evolutionary trajectory of SARS‐CoV‐2, with each new strain potentially exhibiting altered transmissibility, virulence, or immune‐evasion properties [7].

The aim of this study was to assess the extent and composition of the placental inflammatory response in pregnancies complicated by SARS‐CoV‐2 infection and to explore the relationship between SARS‐CoV‐2 RNA levels and the severity of inflammation at delivery using histopathological, ultrastructural, and in situ hybridization analyses.

## 2 Materials and Methods

The research group consisted of 43 placentas derived from 43 women who gave birth at a single tertiary medical center between August 2020 and October 2022 and tested SARS‐CoV‐2‐positive at the time of delivery. Maternal and fetal clinical data were collected retroactively from the electronic medical records.

The study was approved by the local Institutional Review Board. The need for patient consent was waived because of the retrospective design of the study.

### 2.1 Histology

Placental samples were taken according to the criteria of the Amsterdam Placental Workshop Group [8]. The tissues had been fixed in 10% formalin and submitted for standard routine paraffin processing. Slices were stained with hematoxylin and eosin for basic and diagnostic histologic evaluation by two licensed pathologists.

### 2.2 Immunohistochemistry

The formalin‐fixed, paraffin‐embedded tissues (FFPE) were cut into 5 um‐thick slices. The slices were deparaffinized, rehydrated, treated for antigen retrieval.

Immunohistochemical staining was performed using an HRP‐conjugated compact polymer system with DAB chromogen for the following markers: CD3 (Dako, A0452, 1:100; citrate buffer pH 6.0, 95°C−100°C for 20–30 min; Positive Control: human tonsil), CD20 (Dako, L26, M0755, 1:400; citrate buffer pH 6.0, room temperature for 30–60 min; Positive Control: human lymph node), and CD68 (Dako, PG‐M1, M0876, 1:150; citrate buffer pH 6.0, 110°C pressure cooker for 15–30 min; Positive Control: human tonsil).

### 2.3 Transmission Electron Microscopy

In 8 cases, tissues that had been primarily fixed in 10% phosphate‐buffered formalin were processed to wax according to the standard histological protocol. A portion was removed from the wax, chopped into 1 mm cubes, dewaxed in xylene, and rehydrated through graded alcohols to distilled water. This was followed by post‐fixation in 2% aqueous osmium tetroxide and dehydration through a graded series of alcohols and acetone. The ultrathin slices were then embedded in TAAB EMIX medium‐grade epoxy resin (TAAB Laboratories Equipment, Berks, England).

Following heat polymerization for 16 h at 65°C, resin blocks were cut into 0.6 µm sections with a Reichert‐Jung Ultracut E ultramicrotome, stained with 1% toluidine blue in 1% sodium tetraborate, and examined under a light microscope. The most appropriate block was selected and cut into ultrathin sections of 85 nm using a DiATOME diamond knife (DiATOME, Quakertown, PA). The sections were stained with saturated uranyl acetate in 99% ethanol and Reynolds lead citrate.

Sections were examined with a Philips 400 transmission electron microscope equipped with an AMT 16 megapixel mid‐mount digital camera (XR16; Advanced Microscopy Techniques, Woburn, MA). Images were taken in TIF format and converted to JPG with minimum compression.

### 2.4 Quantitative Reverse Transcription Polymerase Chain Reaction (qRT‐PCR)

#### 2.4.1 Total RNA Extraction

To extract total RNA from the FFPE placental samples, sections of the samples were cut at a thickness of 10 µm. Deparaffinization was performed using QIAGEN Deparaffinization Solution (19093,Qiagen, Hilden, Germany), with incubation at 56°C for 3 min followed by cooling at room temperature. The deparaffinized sections were incubated in proteinase K digestion buffer (Buffer PKD) at 56°C for 15 min and subsequently at 80°C for 15 min. Following lysis, the sample underwent centrifugation to remove insoluble debris. The supernatant was treated with DNase I to eliminate any contaminating genomic DNA. Binding conditions were then adjusted with red blood cell buffer (Buffer RBC) and ethanol, and the sample was applied to a RNeasy MinElute spin column (74204, Qiagen, Hilden, Germany). After washing steps with RNA pre‐elution buffer (Buffer RPE), RNA was eluted with RNase‐free diethyl pyrocarbonate (DEPC)‐treated water.

#### 2.4.2 Real Time Reverse Transcription Polymerase Chain Reaction (RT‐PCR) for Variant Detection

Real time RT‐PCR was performed with Allplex SARS‐CoV‐2 Variants I Assay (RV10248X, Seegene Inc., Bothell, WA) according to the manufacturer's protocol. Briefly, extracted RNA (5 ul) was transferred to 96‐well PCR plates containing 15 ul of Master Mix. The plates were spun at 2500 rpm for 5 s and analyzed on a CFX96 Touch Real‐Time PCR (BioRad, Hercules, CA). Parameters for the first reverse transcription reaction cycle were 50°C/ 20 min, 95°C/15 min; and for 45 PCR reaction cycles: 94°C/15 s, 58°C/30 s. Gene amplifications were analyzed by FAM (E484K mutation in the S‐gene), HEX (RdRP), Cal Red 610 (N501Y mutation in the S‐gene), Quasar 705 (69‐70del in the S‐gene), and Quasar 670 (human endogenous internal control) fluorophores. Results were compiled and analyzed using the 2019‐nCoV viewer (Seegene Inc.) according to the manufacturer's instructions.

### 2.5 Statistical Analysis

Data completeness and validity were evaluated, and missing fields were resolved by secondary review of patient documentation. Calibration models were generated using linear regression to construct standard curves. The fit was strong (*R^2^* = 0.94, adjusted *R^2^* = 0.93, residual standard error = 0.19, *F* = 94.1, *p* < 0.001), supporting the reliability of the calibration procedure. Key variables were recoded into clinically meaningful categories (e.g., gestational age at birth categorized as term vs. preterm, placental weight percentiles converted into ordinal categories). Patient demographics and clinical characteristics were summarized as counts (percentages) for categorical variables and as means with standard deviations or medians with interquartile ranges for continuous variables, according to distribution. Group comparisons were conducted using χ^2^ tests (or Fisher's exact test when expected cell counts were <5) for categorical variables, and two‐sample t‐tests or Wilcoxon rank‐sum tests for continuous variables, as appropriate. Normality and distributional assumptions were assessed using histograms, Q–Q plots, and descriptive statistics. All analyses were planned a priori. As the analyses did not involve multiple comparisons, adjustment for false discovery rate was not required. Statistical significance was defined as two‐sided *p* <0.05. All analyses were performed using R version 4.5.1 (R Foundation for Statistical Computing) in RStudio version2025.09.0 (Build 387).

## 3 Results

**FIGURE 1:**
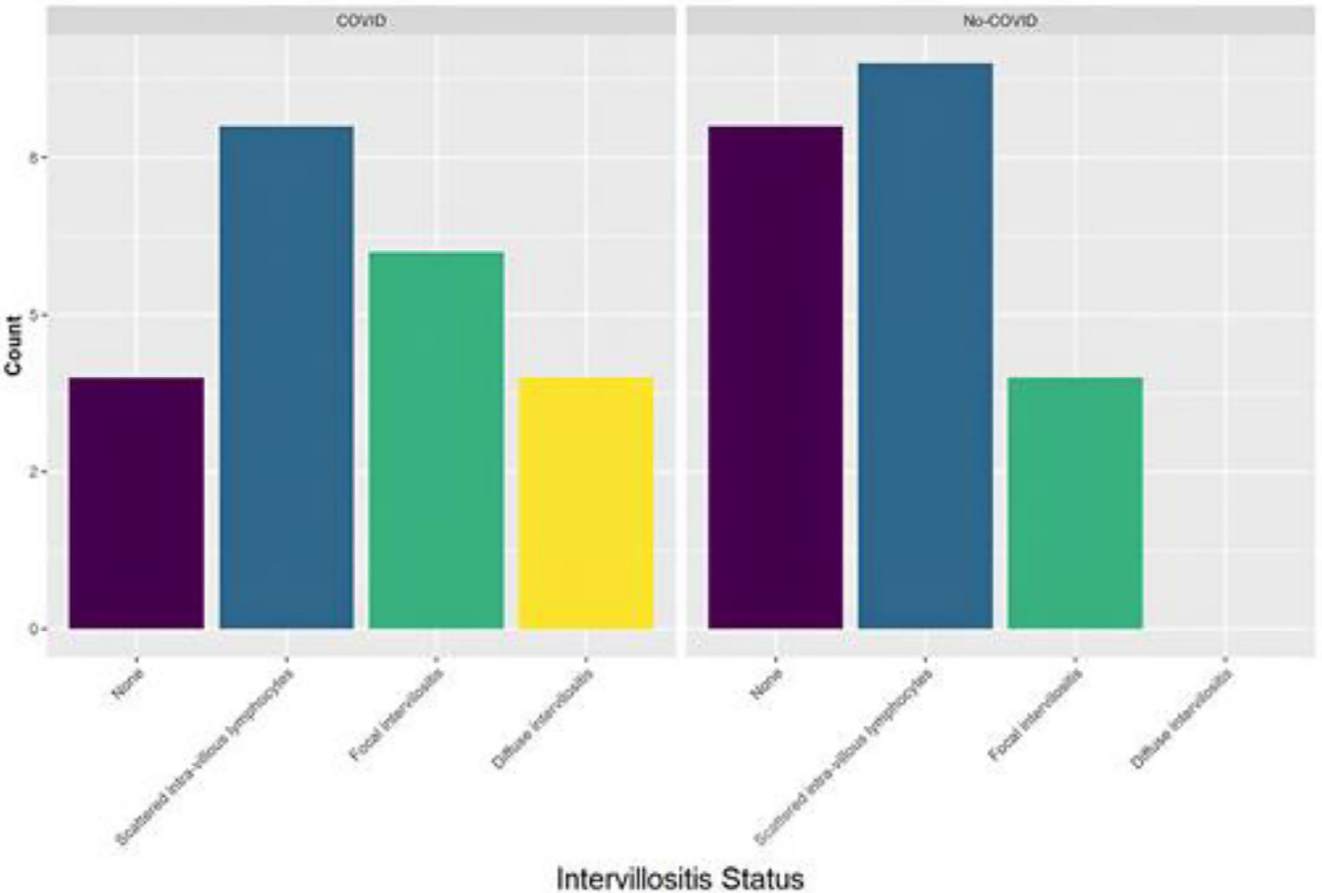
Analysis of PCR‐positive and PCR‐negative placentas by degree of inflammation.

The results are presented in Table 1. Placentas were divided into PCR‐positive and PCR‐negative groups, and each group was further divided into 4 subgroups by severity of CHI (Figure 1): diffuse CHI (*n* = 4), focal CHI (*n* = 10), scattered inflammatory cells (*n* = 17), and no inflammation (*n* = 12).

**TABLE 1:** Characteristics of research group by degree of chronic intervillositis (CHI).

	SARS‐CoV‐2 PCR‐positive					SARS‐CoV‐2 PCR‐negative				
Parameter	Diffuse CHI (*n* = 4)	Focal CHI (*n* = 6)	Scattered cells (*n* = 8)	None (*n* = 4)	*p* value	Diffuse CHI (*n* = 0)	Focal CHI (*n* = 4)	Scattered cells (*n* = 9)	None (*n* = 8)	*p* value
Pt. age (yr)	34.8 ± 4.5	33.7 ± 7.3	30.3 ± 5,8	32.5 ± 3.3	0.5	NA	27 ± 6	31 ± 6	32 ± 7	0.4
Placental weight (g)	447 ± 120	367. ± 143	496 ± 91	371 ± 144	0.13	NA	530 ± 86	455 ± 112	469 ± 129	0.6
Gestational age (wk)	37.0 ± 0.8	34.3 ± 4.1	34.75 ± 3.7	32.75 ± 6.7	0.7	NA	38.75 ± 2.63	37.3 ± 1.9	37.38 ± 0.92	0.4
Birth weight										
LGA	0	0	2 (25%)	0	0.5	NA				
Normal	4 (100%)	5 (83%)	6 (75%)	4 (100%)			4 (100%)	9 (100%)	7 (88%)	
SGA	0	1 (17%)	0	0			0	0	1 (13%)	
Term birth	3 (75%)	3 (50%)	2 (25%)	2 (50%)	0/4	NA	3 (75%)	6 (67%)	7 (88%)	0.8
E484K mutation^a^	0	0	0	0		NA	0	0	0	
RdRp gene^b^	4 (100%)	6 (100%)	7 (88%)	4 (100%)	>0.9	NA	0	0	0	
N501Y mutation^a^	2 (50%)	2 (33%)	3 (38%)	1 (25%)	>0.9	NA	0	0	0	
69/70 deletion^a^	1 (25%)	2 (33%)	0	1 (25%)	0.3	NA	0	0	0	
Viral RNA copies	1156 ± 1378	222 ± 523	1 ± 0	1 ± 1	0.39	NA	NA	NA	NA	
CD20 marker	4 (100%)	5 (83%)	5 (63%)	0	0.019	NA	4 (100%)	4 (44%)	0	0.002
Maternal inflammation	3 (75%)	5 (83%)	4 (50%)	2 (50%)	0.6	NA	2 (50%)	6 (67%)	4 (50%)	0.9
Fetal inflammation	1 (25%)	2 (33%)	0	1 (25%)	0.3	NA	3 (75%)	2 (22%)	0	0.022
Pre‐eclampsia	1 (25%)	0	0	0	0/4	NA	0	0	0	

*Notes:* Values are presented as mean ± SD or n(%). Analyses were performed with Kruskal–Wallis and Fisher exact tests.

Abbreviation: NA, not applicable

^a^ Mutations in the spike protein of SARS‐CoV‐2 virus

^b^ RNA‐dependent RNA polymerase gene

PCR was positive in 22/43 placentas: all 4 with diffuse intervillositis (mean viral load, 1155.57/mL), including one with preeclampsia; 6/10 with focal intervillositis (mean viral load 221.91/mL); 8/17 with scattered inflammatory cells (mean viral load, 0.79/mL), and 4/12 with no inflammation (mean viral load, 1.05/mL).

**FIGURE 2:**
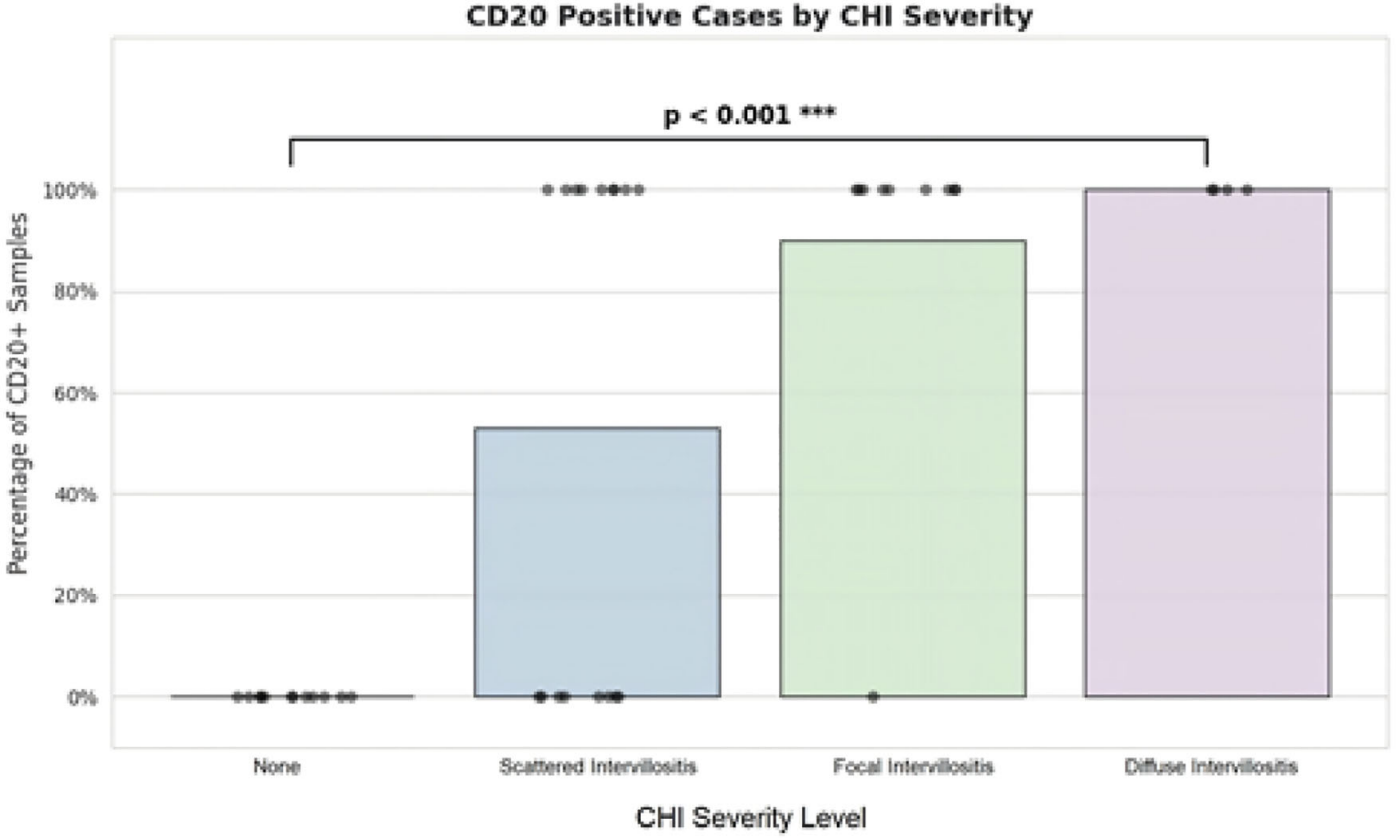
Quantity of CD20‐positive lymphocytes in PCR‐positive and PCR‐negative placentas.

There was a statistically significant association between viral load and severity of infection. Overall, maternal inflammatory conditions (lymphoplasmacytic deciduitis and acute chorioamnionitis) were found in 14 cases, and fetal inflammatory conditions (chronic nonspecific villositis), in 4 cases. **The prevalence of coincident maternal inflammatory conditions** was most pronounced in the diffuse intervillositis subgroup, reaching 75%, and of **coincident fetal inflammatory conditions**, in the focal intervillositis subgroup, reaching 50%. The rate of coincident fetal inflammation was low, only 25%, in the presence of diffuse intervillositis, probably because of the small size of the subgroup. CD20‐positive lymphocytes were identified in 14 cases. The prevalence of CD20‐positive lymphocytes was highest (100%) in the diffuse intervillositis subgroup, and lowest (0%) in the subgroup with no inflammation (Figure 2).

PCR was negative in 21/43 patients, of whom 4 had focal intervillositis and 9 had scattered inflammatory cells. The rest were negative for any signs of intervillositis. None had diffuse intervillositis. There were 12 cases of associated maternal inflammation, and 5 cases of associated fetal inflammation. CD20‐positive lymphocytes were identified in 9 PCR‐negative placentas (Figure 2).

The placental weights fell largely (90.7%) within the normal range for respective gestational age.

**FIGURE 3:**
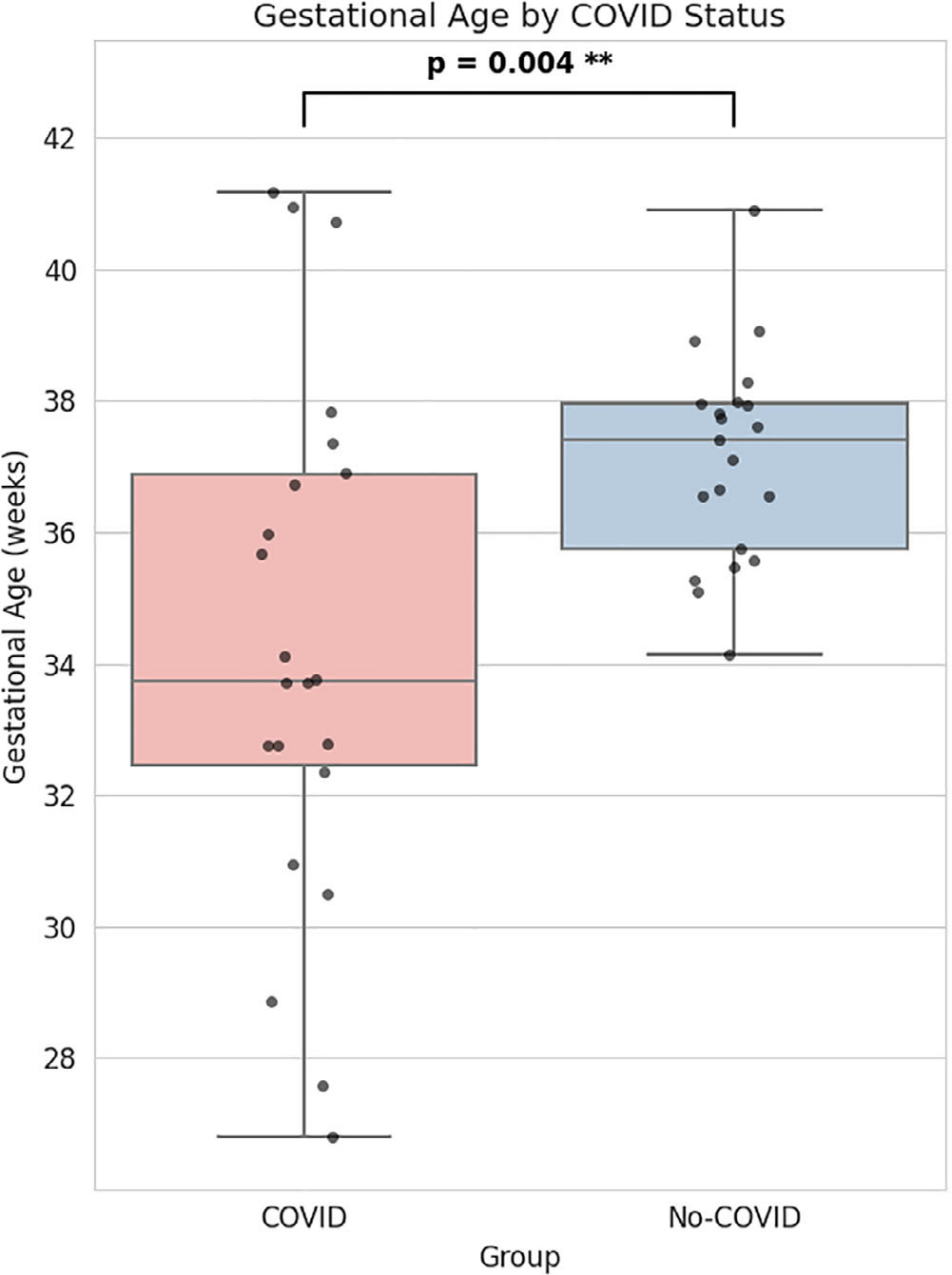
Mean gestational age by PCR status.

Mean gestational age was significantly lower in the PCR‐positive group than the PCR‐negative group (34.68 weeks vs 37.62 weeks, *p* = 0.004) (Figure 3).

**FIGURE 4:**
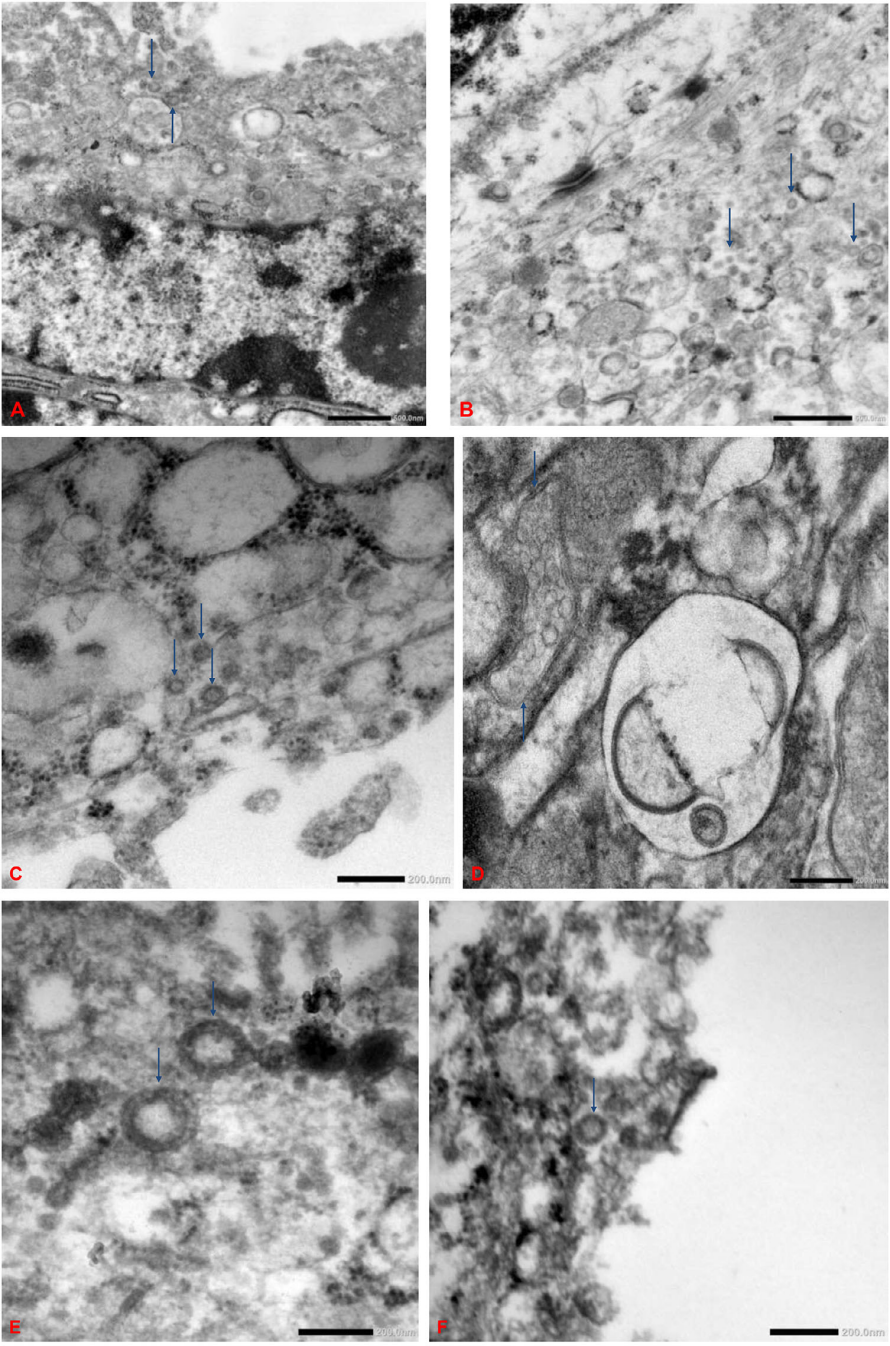
Electron microscopy image of formalin fixed‐paraffin samples. Note the multiple SARS‐CoV‐2 virions lying free and within a vacuole (blue arrows). (A) PCR‐positive placenta with diffuse intervillositis. (B) PCR‐positive placenta with focal intervillositis. (C) PCR‐negative placenta with focal intervillositis. (D) PCR‐positive placenta without any inflammatory cells. (E) PCR‐positive placenta with scattered inflammatory cells. (F) PCR‐negative placenta with scattered inflammatory cells.

Electron microscopy was conducted in 8 placentas (Figure 4). Six were PCR‐positive, among which 2 exhibited diffuse CHI, 1 focal CHI, 2 scattered inflammatory cells, and 1 no intervillositis. The other 2 placentas were PCR‐negative; one exhibited focal intervillositis, and one, scattered inflammatory cells. Viral particles, located primarily in the syncytiotrophoblasts, measured 87 to 178 nm in diameter and featured spikes of approximately 17 nm, resembling SARS‐CoV‐2.

## 4 Discussion

Transmission of SARS‐CoV‐2 into and across the placenta critically depends on the availability and functionality of the viral entry mechanisms in the placental syncytiotrophoblast layer. Cellular entry of SARS‐CoV‐2 is facilitated by its surface‐anchored spike protein (S) which binds to the angiotensin‐converting enzyme 2 (ACE2) receptor, triggering the initial attachment. Further cell entry steps include activation of the virus internalization process by host cell proteases, in particular cell‐surface transmembrane protease, serine 2 (TMPRSS2) and lysosomal cathepsin proteases, fusion of viral and cellular membranes, and endocytosis [9].

CHI was known to affect the placentas of pregnant women also in the pre‐pandemic era, but the histological picture differed. Pre‐pandemic CHI was characterized by the presence of a uniform population of monocyte‐macrophages at varying stages of maturity and activation: more than 90% CD45‐ and CD68‐positive, 30% to 40% MAC387‐positive, less than 5% CD3‐positive, and CDla‐, CD20‐, CD30‐, and CD56‐negative [10]. By contrast, in SARS‐CoV‐2‐associated CHI, specific distinct CD20+ B cells have also been reported [4, 11]. The incidence of pre‐pandemic CHI was very low (<1% of all pregnancies), and it was presumed to have an immunological basis, to carry a very high recurrence risk, and to be associated with intrauterine growth restriction [3].

Particular attention has been addressed to the presence of CD20‐positive B lymphocytes, which are known to migrate to sites of inflammation, especially during viral infections. B cells have the multifaceted ability to produce antibodies, interact with T cells, and respond to antigenic stimuli. SARS‐CoV‐2 infection, by altering the B‐cell populations in the placenta, changes its inflammatory landscape. B cells respond to antigenic stimuli by the systemic production of inflammatory cytokines, especially IL‐6 which is known to be present in elevated levels in severe courses of SARS‐CoV‐2 infection. Moreover, anti‐SARS‐CoV‐2 neutralizing antibodies produced by B lymphocytes, particularly CD20‐positive cells, can potentially impact both systemic and placental inflammation [12]. This premise is supported by studies demonstrating the presence of B lymphocytes in SARS‐CoV‐2‐associated CHI and their direct correlation with the severity of the condition [5]. Our results are consistent with these findings, confirming the relationship between increased CD20‐positive B cells and disease severity [13]. Nevertheless, it remains unclear why intervillositis develops in some SARS‐CoV‐2‐positive mothers but not others.

In contrast to previous studies [3, 14, 15], our cohort demonstrated over 50% positivity for viral RNA in placental tissue. Viral loads correlated with the severity of CHI, presence of CD20‐positive B lymphocytes, and concomitant maternal and fetal inflammation.

Among the 22 placentas that tested PCR‐positive, 4 were negative for CHI, and 8 demonstrated only scattered inflammatory cells, possibly indicative of inefficient viral replication [15]. Conversely, 4 of the 21 placentas that tested PCR‐negative exhibited focal CHI with CD20‐positive cells, while the remaining specimens either presented with dispersed inflammatory cells (9 cases) or had no observable inflammation (8 cases). Interestingly 4 of the 9 PCR‐negative cases with scattered inflammatory cells had CD20‐positive lymphocytes (Figure 2). This variability may suggest that the virus can induce an inflammatory response even when present in undetectable levels. It is further supported by the electron microscopy studies which revealed the presence of the viral particles within the syncytiotrophoblasts of both the PCR‐positive and PCR‐negative placentas, indicating that the virus can replicate at levels undetectable by PCR. Together, these findings highlight the potential of the virus to persist and impact placental function without being identified through conventional diagnostic methods.

The significantly lower mean gestational age in the PCR‐positive group than the PCR‐negative group indicates that SARS‐CoV‐2 infection may be associated with earlier deliveries (Figure 3).

### 4.1 Study Limitations

The lack of data on maternal symptoms at the time of delivery limited our ability to correlate maternal health status with placental findings. Additionally, data were lacking on fetal outcomes, which would have provided a valuable context for interpreting the placental findings. Understanding the relationship between maternal symptoms, placental health, and fetal outcomes is crucial for a comprehensive analysis.

The samples were not tested for any other bacterial or viral infection. However, CHI is not known to be associated with particular pathogen and has been **referred to have** an immunologic etiology. Moreover, the scope of this research was limited by the small cohort (*n* = 43), potentially affecting the statistical power of the analysis. PCR sensitivity in formalin‐fixed and paraffin‐embedded (FFPE) samples is primarily limited by DNA/RNA degradation, cross‐linking, and inhibitory substances, while viral RNA is relatively stable within tissue.

## 5 Conclusions

This study shows that CHI can occur even in PCR‐negative placentas, implying that the virus may replicate below the detection threshold of the PCR assay or adversely influence placental health through other mechanisms without direct detection. B lymphocytes are implicated in SARS intervillositis via their role in the production of inflammatory cytokines, particularly IL‐6. The severity of CHI is correlated with the presence of concurrent fetal and maternal inflammation. The reduced gestational age in the presence of a positive PCR test points to a potential risk that the disease could lead to premature births. These findings emphasize the need for further investigations.

The association of viral load with the severity of CHI together with the presence of CD20‐positive B lymphocytes in the inflamed placenta and the concomitant maternal and fetal inflammation (Table 1) suggest that the inflammatory response is influenced by factors beyond just the detectable presence of the virus. The detection of viral particles by electron microscopy in all cases supports the notion of silent replication. Differences in viral load and individual variability in immune responses may be part of the reason the inflammation develops in some individuals but not others. Further research is needed to elucidate these mechanisms and understand their implications for maternal and neonatal health.

Further studies with improved sampling strategies and larger subgroups would increase the generalizability of the findings.

## Funding

This research did not receive any specific grant from funding agencies in the public, commercial, or not‐for‐profit sectors.

## Conflicts of Interest

The authors declare that the research was conducted in the absence of any commercial or financial relationships that could be construed as a potential conflict of interest.
